# A Novel Multi-Source Image Registration of Porcine Body for Multi-Feature Detection

**DOI:** 10.3390/s25226918

**Published:** 2025-11-12

**Authors:** Zhen Zhong, Shengfei Zhi

**Affiliations:** 1 School of Electrical and Information Engineering, Tianjin University, Tianjin 300372, China; 2 Post-Doctoral Workstation, Tianjin Development Zone Zhonghuan System Electronic Engineering Co., Ltd., Tianjin 300074, China; 3 School of Information Technology Engineering, Tianjin University of Technology and Education, Tianjin 300222, China

**Keywords:** visible and infrared image registration, Gabor-ordinal-based contour angle orientation, modified scale-invariant feature transform, porcine body multi-feature detection

## Abstract

**Highlights:**

**What are the main findings?**

- A novel main orientation representation algorithm of feature points is presented for visible and infrared porcine body images.
- A novel visible and infrared porcine body image registration model is constructed to enhance registration accuracy in variable illumination conditions.

**What is the implication of the main finding?**

- The visible and infrared porcine body image registration method can achieve a lower average root-mean-square error than current registration algorithms.

**Abstract:**

The safety of animal-related agricultural products has been a hot issue. To obtain a multi-feature representation of porcine bodies for detecting their health, visible and infrared imaging is valuable for exploiting multiple images of a porcine body from different modalities. However, the direct registration of visible and infrared porcine body images can easily cause the dislocation of structural information and spatial position, due to different resolutions and spectrums of multi-source images. To overcome the problem, a novel multi-source image feature representation method based on contour angle orientation is proposed and named Gabor-Ordinal-based Contour Angle Orientation (GOCAO). Moreover, a visible and infrared porcine body image registration method is described and named GOCAO-Rough to Fine (GOCAO-R2F). First, contour and texture features of the porcine body are acquired using a Gabor filter with variable scales and an ordinal operation. Second, feature points in contours are obtained by curvature scale space (CSS), and the main orientation of each feature point is determined by GOCAO. Third, modified scale-invariant feature transform (MSIFT) features are received on the main orientation and registered with bilateral matching. Finally, accurate registrations are extracted by R2F. Experimental results show that the proposed registration algorithm accurately matches multi-source images for porcine body multi-feature detection and is capable of achieving lower average root-mean-square error than current registration algorithms.

## 1. Introduction

As quality of life continues to improve, safety issues in agricultural products, especially those related to animals, have attracted public attention. Porcine body health is vital to the safety of animal-related products, and an effective multi-source porcine body image analysis method can achieve non-contact porcine body health detection.

To achieve accurate detection of porcine body health, weight estimation [1] and behavior detection [2] are used to detect abnormal phenomena in a timely manner. Nowadays, deep learning-based methods are used to fit 2D image features into 3D models to accurately calculate weight and behavioral features [3,4]. However, it is difficult to acquire a large number of 3D models in practice, which reduces the reliability of animal 3D reconstruction. Due to the dependence of porcine body shape features on the construction of 3D models, which is also an important manifestation of animal health, shape feature extraction is crucial for achieving accurate detection of porcine body health. Moreover, the increase in porcine body temperature is one of the earliest adverse health conditions. Therefore, to timely detect the health status of the porcine body, shape and temperature features are considered as porcine body health representation objects. However, it is difficult to obtain porcine body shape and temperature features based on uni-source images, and the direct registration of multi-source porcine body images is prone to causing the dislocation of structural information and spatial position.

**Figure 1:**
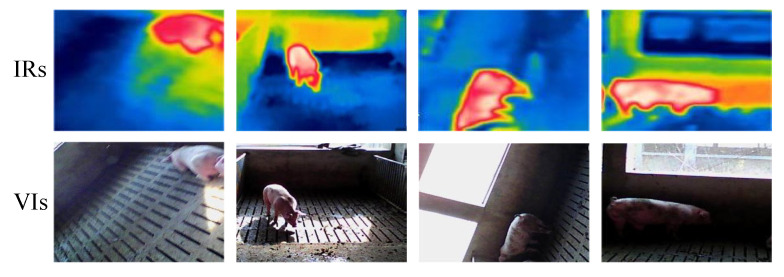
The misalignment phenomenon of multi-source porcine body images.

**Figure 2:**
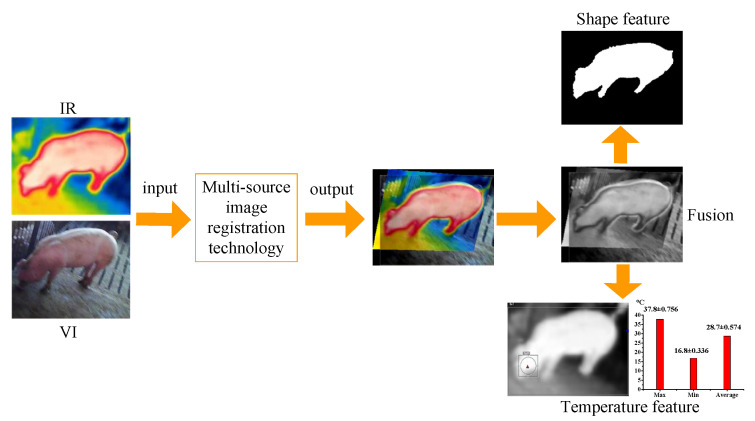
The procedure of porcine body multi-feature detection.

To obtain porcine body shape and temperature features, the multi-source porcine body images are gained via FLIR C2 (FLIR Systems, USA), which can realize simultaneous acquisition of visible and infrared images, and the main technical indicators are shown in Table 1. However, due to the different spectrums and imaging resolutions of thermal infrared detectors and digital cameras, the acquired multi-source images may have a misalignment phenomenon, which seriously affects the multi-source porcine body image fusion and multi-feature detection. The examples of misalignment phenomena are shown in Figure 1. To effectively extract shape and temperature features of the porcine body, visible and infrared image registration is a significant procedure for multi-source image fusion and multi-feature detection, as shown in Figure 2.

The aim of visible and infrared image registration is to align two images, which are captured at multiple viewpoints, modalities, or times. Gray-based methods utilize the local similarity descriptors to optimize the alignment processing [5], which mainly contain methods based on mutual information (MI) [6], mean squared difference (MSD) [7], and cross correlation (CC) [8]. However, the registration methods based on CC have a high complexity, which affects the efficiency of the algorithm and reduces the actual application requirements [9]. Due to the different local gray mapping relationships of multi-source images and the interference of noise, the registration methods based on MI and MSD can lead to low accuracy of algorithms [10,11].

**Figure 3:**
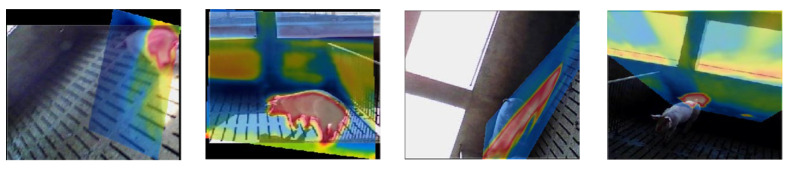
The examples of registered visible and infrared porcine body images based on CAO-C2F.

Feature-based methods extract various features and construct a deformation function to minimize the feature variability between multi-view, multi-source, and multi-time images [12,13]. The most commonly used methods mainly include SIFT [14,15], BRIEF [16], SURF [17,18], and ORB [19]. Due to the obvious variations in multi-source images, the error rate is relatively high in registration processing. To enhance the robustness of multi-modal feature descriptors, a partial intensity invariant feature descriptor (PIIFD) is proposed [20]. However, due to the lack of scale invariance in PIIFD, it is impossible to establish reliable correspondence between visible and infrared porcine body images. To enhance the scale invariance and reduce the influence of different spectrums and resolutions in multi-source images, a contour angle orientation (CAO) method is proposed [21]. However, existing methods cannot meet the requirements of visible and infrared porcine body image registration, especially in the case of variable illumination conditions. As CAO-C2F has been proven to be the most accurate method for registering multi-source images among four state-of-the-art methods, which contain SIFT-LPM, SI-PIIFD-LPM, EG-SURF-RANSAC, and CAO-C2F [21], especially in multi-source images with obvious contour features, the examples of registered visible and infrared porcine body images based on CAO-C2F are shown in Figure 3. It can be seen that multi-source porcine body images are not effectively registered by CAO-C2F, especially in the case of poor illumination.

**Figure 4:**
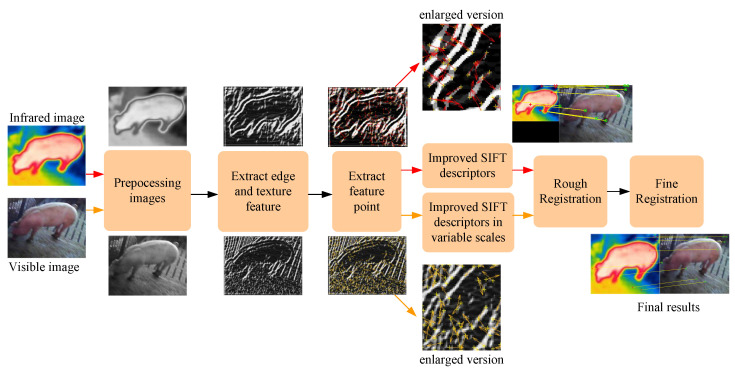
The flowchart of the proposed visible and infrared porcine body image registration framework.

Nowadays, deep learning-based methods have great advantages in multi-source image registration, but they require a large amount of training data and real registered values [22]. Therefore, considering the lack of real value in multi-source registration, a novel visible and infrared porcine body image registration algorithm is proposed and named GOCAO-Rough to Fine (GOCAO-R2F), which is demonstrated in Figure 4.

The main superiorities of the proposed visible and infrared image registration algorithm are summarized as follows:

- A novel main orientation representation algorithm of feature points is presented for visible and infrared porcine body images.
- A novel visible and infrared porcine body image registration model is constructed to enhance registration accuracy in variable illumination conditions.
- The visible and infrared porcine body image registration method can achieve a lower average root-mean-square error than current registration algorithms.

## 2. Materials and Methods

In this section, a visible and infrared porcine body image registration algorithm is presented in detail. First, the proposed visible and infrared image registration algorithm is provided to obtain reliable multi-source porcine body images. Second, the multi-feature is represented in view of multi-source porcine body fusion images.

### 2.1. Multi-Source Porcine Body Image Registration

#### 2.1.1. Gabor-Ordinal-Based Contour Angle Orientation

To achieve spectrum and resolution invariance of visible and infrared porcine body images, a novel multi-source image representation algorithm is proposed and named Gabor-Ordinal-based Contour Angle Orientation (GOCAO).

**Figure 5:**
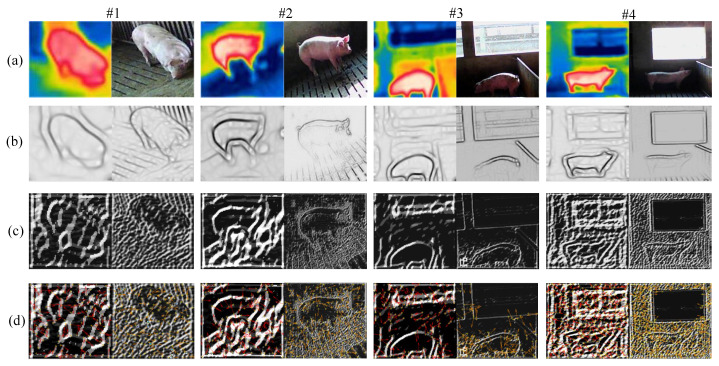
Sketch maps of contour feature under variable illumination conditions: (**a**) porcine body infrared and visible images; (**b**) Gabor-based porcine body contour feature maps; (**c**) Gabor-Ordinal-based porcine body contour feature maps; (**d**) Gabor-Ordinal-based contour angle orientation feature maps.

First, due to the adjustable center frequency and orientation of the Gabor filter [23], the porcine body feature maps are acquired by the Gabor filter with variable scales, which can effectively represent the porcine body contour features, and the sketch maps of the Gabor-based contour feature *F*(*x*,*y*) in multi-source porcine body images with variable illumination conditions are shown in Figure 5b.

(1) $$G(x,y)=\frac{\gamma }{2\pi \sigma^{2}}\exp\{-\frac{1}{2}(\frac{x_{\theta }^{2}+\gamma^{2}y_{\theta }^{2}}{\sigma^{2}})\}\cos(\hat{j}2\pi fx_{\theta })$$

(2) F(x,y)=I(x,y)⊗G(x,y)

where *G*(.) and *I*(.) represent an even-symmetry Gabor filter and multi-source porcine body images, respectively. *f* and *θ* are the center frequency and angle, respectively. *σ* and *γ* are the scale and length–-width ratio of the envelope, respectively. ⊗ is a convolution operation. *x_θ_* and *y_θ_* are angles in the *x* and *y* directions, which are shown as follows:

(3) $$\left\{\begin{array}{l} x_{\theta }=x\cos\theta +y\sin\theta ,\\ y_{\theta }=-x\sin\theta +y\cos\theta . \end{array}\right.$$

Second, to improve the robustness and richness of contour feature extraction, the ordinal features are obtained by the Ordinal filter with triple lobes [24]. The sketch maps of Gabor-Ordinal-based contour feature *S* in multi-source porcine body images with variable illumination conditions are shown in Figure 5c.

(4) $$Ordinal(x,y)=C_{p}\sum_{i=1}^{N_{p}}\frac{1}{\sqrt{2\pi \sigma_{pi}}}\exp[\frac{-{\left(F(x,y)-\omega_{pi}\right)}^{2}}{2\sigma_{pi}^{2}}]-C_{n}\sum_{j=1}^{N_{n}}\frac{1}{\sqrt{2\pi \sigma_{nj}}}\exp[\frac{-{\left(F(x,y)-\omega_{nj}\right)}^{2}}{2\sigma_{nj}^{2}}]$$

(5) $$S=F(x,y)⊗Ordinal(x,y)=\{\Gamma^{j}|\Gamma^{j}={\left\{P_{1}^{j},P_{2}^{j},\ldots ,P_{n}^{j}\right\}}_{j=1}^{N_{s}}\}$$

where *ω* and *δ* are the central position and scale of the ordinal filter, respectively. *N_p_* and *N_n_* are the number of positive and negative lobes, respectively. Constant coefficients *C_n_* and *C_p_* are adopted to maintain a balance between positive and negative lobes. *F*(.) represents Gabor-based contour feature maps, *Γ^j^* is the jth contour feature in *S*, and *N_s_* is the number of contour features in *S*. *n* represents the number of pixels in each contour, *P^j^* represents a pixel in the *j*th contour feature, and each pixel is viewed as a feature point in this paper.

**Figure 6:**
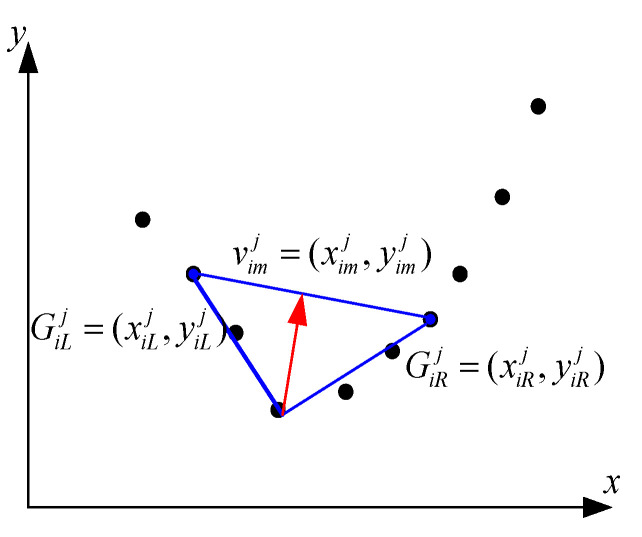
Sketch map of computing the orientation of the Gabor-Ordinal-based feature point.

Next, the feature points of visible and infrared porcine body images are represented by curvature scale space (CSS) [25] and assumed as *G_iL_^j^* = (*x_iL_^j^*, *y_iL_^j^*) and *G_iR_^j^* = (*x_iR_^j^*, *y_iR_^j^*), respectively. The orientation of the angular bisector vector *v_im_^j^* is displayed in Figure 6, and it can be defined as follows:

(6) $$v_{im}^{j}=(x_{im}^{j},y_{im}^{j})=\min(||(x_{iL}^{j},y_{iL}^{j})|{|}_{2},||(x_{iR}^{j},y_{iR}^{j})|{|}_{2})(\frac{\left(x_{iL}^{j},y_{iL}^{j}\right)}{||(x_{iL}^{j},y_{iL}^{j})|{|}_{2}}+\frac{\left(x_{iR}^{j},y_{iR}^{j}\right)}{||(x_{iR}^{j},y_{iR}^{j})|{|}_{2}})$$

where *x_im_^j^* and *y_im_^j^* represent the horizontal and vertical orientation of the *j*th angular bisector vector in the *i*th pair of multi-source porcine body images, respectively. *x_iL_^j^* and *y_iL_^j^* represent the horizontal and vertical orientation of the *j*th angular bisector vector in the *i*th visible porcine body image, respectively. *x_iR_^j^* and *y_iR_^j^* represent the horizontal and vertical orientation of the *j*th angular bisector vector in the *i*th infrared porcine body image, respectively.

Finally, the Gabor-Ordinal-based contour angle orientation of each feature point is indicated as follows:

(7) $$O(Ordinal_{i}^{j})=\left\{\begin{array}{l} \tan^{-1}(\frac{y_{im}^{j}}{x_{im}^{j}}),\;y_{im}^{j}\geq 0\cap x_{im}^{j}\geq 0\\ \tan^{-1}(\frac{y_{im}^{j}}{x_{im}^{j}})+\pi ,\;x_{im}^{j}<0\;\\ \tan^{-1}(\frac{y_{im}^{j}}{x_{im}^{j}})+2\pi ,\;y_{im}^{j}<0\cap x_{im}^{j}>0 \end{array}\right.i=1,2,\ldots ,n;\;j=1,2,\ldots ,N_{s}$$

where *x_im_^j^* and *y_im_^j^* represent the horizontal and vertical orientation of the *j*th angular bisector vector in the *i*th pair of multi-source porcine body images, respectively. *Ordinal_i_^j^* represents the *j*th feature point in the *i*th Gabor ordinal feature map. Moreover, the Gabor-Ordinal-based contour angle orientation feature maps are viewed in Figure 5d.

#### 2.1.2. Multi-Source Porcine Body Feature Rough to Fine Registration

Due to the scale and rotation invariance of SIFT, modified SIFT (MSIFT) is adopted to improve the grayscale invariance of multi-source Gabor-Ordinal-based porcine body feature images [26], the gray gradient magnitudes of which are normalized to lower the interference of different spectrums, and the orientation histogram dimension with 16 bins is converted to an orientation histogram with only eight bins. Considering the rich scale information of visible porcine body images, multi-scale MSIFT features are received on the main orientation, which is determined by GOCAO. Moreover, the threshold of MSIFT is chosen empirically according to different illuminations.

**Figure 7:**
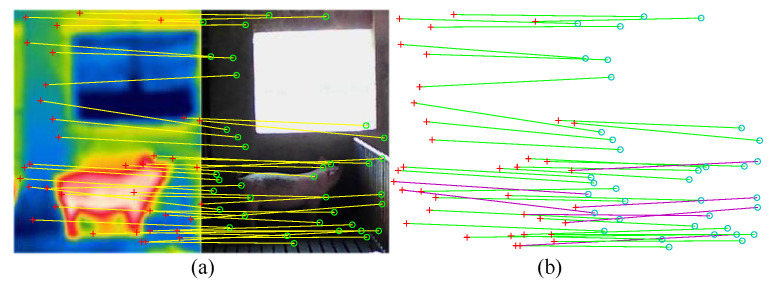
Sketch map of rough registration: (**a**) rough registration result; (**b**) possible correct matches in green lines and identified mismatches in purple lines.

Then, the random sample consensus (RANSAC) algorithm [27] is adopted to estimate scale parameters, which can omit obvious incorrect matches. The registered visible and infrared porcine body feature points of rough registration are required and denoted as P_ri_ and P_rv_, respectively, which is shown in Figure 7.

**Figure 8:**
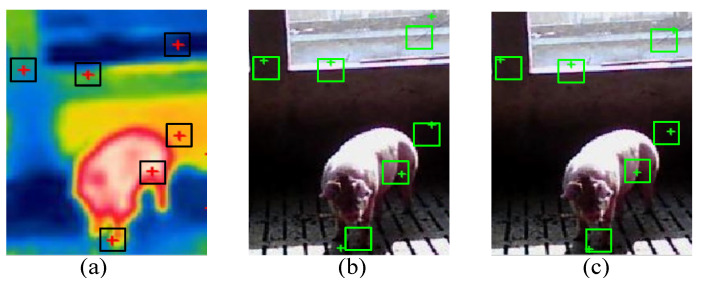
Feature points location of fine registration: (**a**) Infrared registration. (**b**) Visible rough registration. (**c**) Visible fine registration.

Finally, due to different resolutions of multi-source Gabor-Ordinal-based porcine body feature images, the spatial positions of feature points have deviations. To overcome this problem, fine matches can be obtained by the proposed method in [9], which can reduce the deviation of rough matches, and the location of P_rv_ is renewed. The sketch map is shown in Figure 8. It can be seen that the location errors are corrected to the right position based on fine registration.

### 2.2. Porcine Body Multi-Feature Representation

To test the effectiveness of visible and infrared image registration, a porcine body multi-feature representation method is adopted in view of a multi-source image fusion method, which is my previously proposed method [28].

## 3. Results

**Figure 9:**
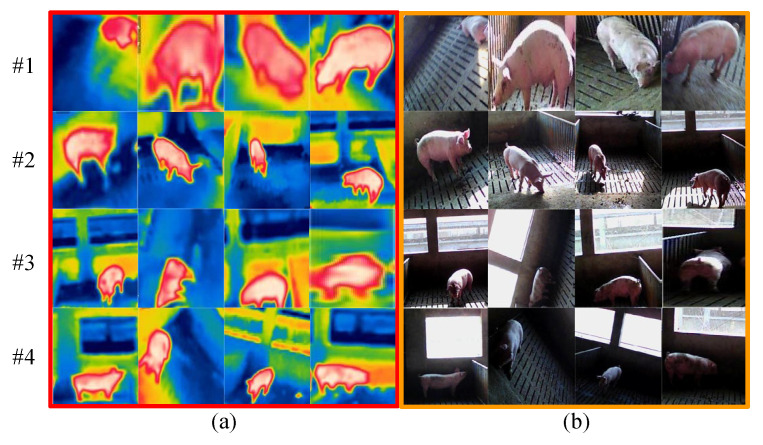
Sketch maps of self-collected database: (**a**) infrared porcine body images; (**b**) visible porcine body images.

The proposed visible and infrared porcine body registration model is achieved by MATLAB (v2014a) on a notebook with a 2.6 GHz Intel Core CPU and an 8 GB RAM. To estimate the performance of the proposed multi-source image registration algorithm, a self-collected multi-source porcine body database is built by FLIR C2 and revealed in Figure 9. It can be seen that visible and infrared images are divided into four groups according to the intensity of visible porcine body images with variable illumination conditions, which are represented as #1, #2, #3, and #4.

To verify the registration performance of the proposed algorithm, five state-of-the-art visible and infrared image registration methods, such as Dense [29], CAO-C2F [21], SI-PIIFD-RPM [20], EG-SURF-RANSAC [30], and SIFT-LPM [31], are compared to the proposed method. Moreover, objective evaluation metrics are commonly adopted to estimate the registration and detection results, which are root-mean-square error (*RMSE*) [32], Precision–Recall [33], and Accuracy [34].

*RMSE* is the root-mean-square error between experimental registration points acquired through registration methods and reference registration points acquired through manual calibration; its formula is as follows:

(8) $$RMSE=\sqrt{\frac{\sum_{i=1}^{N}||(x_{i},y_{i})-(x_{i}^{ref},y_{i}^{ref})|{|}_{2}}{N}}$$

where (*x_i_*,*y_i_*) and (*x_i_^ref^*,*y_i_^ref^*) are the coordinates of the *i*th matching point and reference matching point, respectively. *N* is the number of final registration points, which is acquired via R2F matching. The smaller the *RMSE*, the higher the registration accuracy.

*Precision* is the ratio of the number of correct matches to the total number of matches, both of which are acquired through the registration method, whose formula is as follows:

(9) $$precision=\frac{correct\;matches}{total\;matches}$$

where *total matches* contain correct matches and false matches. *Correct matches* are the number of those matches that satisfy

(10) $$||(x_{i},y_{i})-(x_{i}^{ref},y_{i}^{ref})|{|}_{2}\leq 5$$

*False matches* are the rest of the total matches. The distance threshold is chosen empirically. The higher the *precision*, the more accurate the feature registration method.

*Recall* is the ratio of the number of correct matches to the number of correct matches in the feature points described by CSS, which represents the ability of the registration method to obtain correct matches from multi-source porcine body images taken under variable illumination conditions. Its formula is as follows:

(11) $$recall=\frac{correct\;matches}{correspond\;features}$$

where correspond features represent the number of *correct matches* in the feature points described by CSS. The larger the *recall*, the better the ability of the registration algorithm to distinguish feature points.

*Accuracy* is used to measure the detection rate of the porcine body shape feature and is defined as follows:

(12) $$Accuracy=\frac{TP+TN}{TP+TN+FP+FN}$$

where *TP* and *TN* are true positives and negatives, respectively. *FP* and *FN* are false positives and negatives, respectively. The better the *accuracy*, the more accurate the porcine body shape feature detection method.

### 3.1. Comparisons of Main Orientation

The main orientation is a major property of Gabor-Ordinal-based feature points in the procedure of visible and infrared porcine body image registration algorithm. The more correct matches with the same main orientation, the more precise the final matches will be. In this section, the absolute error of the angle is less than 5°.

**Figure 10:**
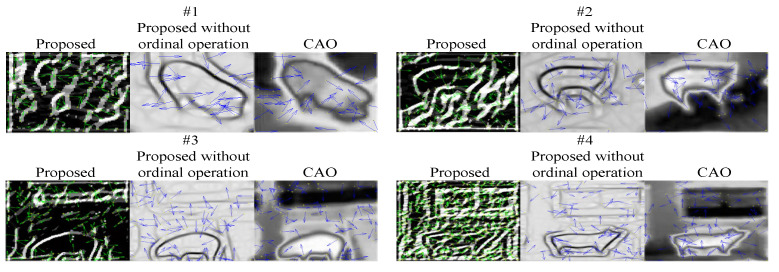
The comparisons of the main orientation.

To prove the superior performance of the GOCAO, it is compared with CAO, which has been proven to have the best representation of the main orientation among four state-of-the-art visible and infrared image registration methods [21]. The four sketch maps are composed of one image chosen under four variable illumination conditions, and the results are shown in Figure 10. It can be seen that the number of main orientations of feature points extracted by the proposed method is significantly more than that by CAO, and it can be further explained that the number of extracted feature points is maximum based on the proposed method. Therefore, it can lay the foundation for feature matching based on multi-source porcine body images.

### 3.2. The Registration Performance of the Proposed Method

**Figure 11:**
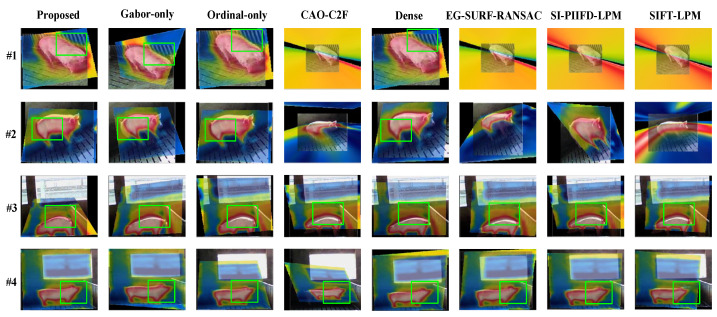
The registration results based on different multi-source image registration methods with variable illumination conditions.

To achieve qualitative analysis of registration performance, four multi-source porcine body image pairs are composed of one image pair, which was chosen under four variable illumination conditions, and the registration results are shown in Figure 11. It can be seen that the proposed method can effectively register contour features in visible and infrared porcine body images, especially in poor illumination. Moreover, the results reflect the contributions of Gabor filtering and the Ordinal operation.

**Figure 12:**
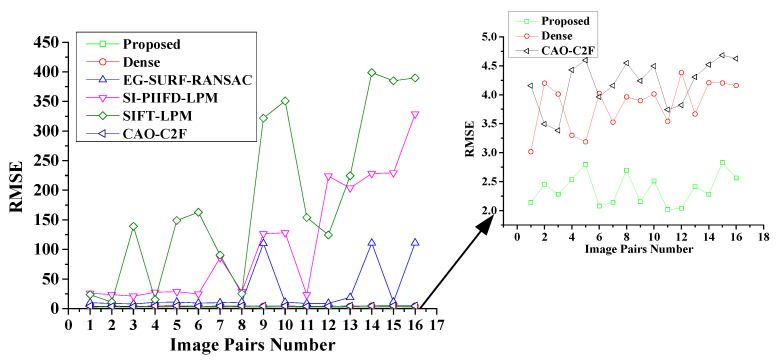
The RMSE of the six considered methods with variable illumination conditions.

To conduct a quantitative analysis of the registration performance, the RMSEs of the considered methods are shown in Figure 12. The GOCAO-R2F fulfils the minimum RMSE among the six considered registration methods. Due to the smaller RMSE and the better registration performance, five state-of-the-art visible and infrared image registration methods fail to effectively align most of the porcine body images with variable illumination conditions, except the proposed multi-source image registration method.

**Figure 13:**
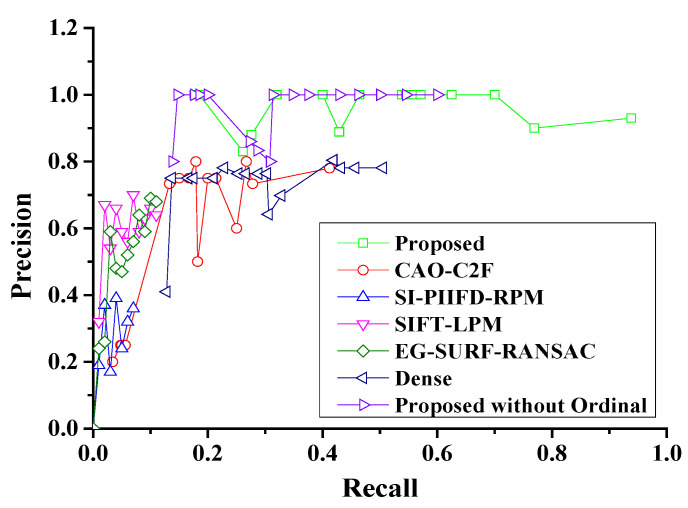
The Precision–Recall curves of the seven considered algorithms.

**Figure 14:**
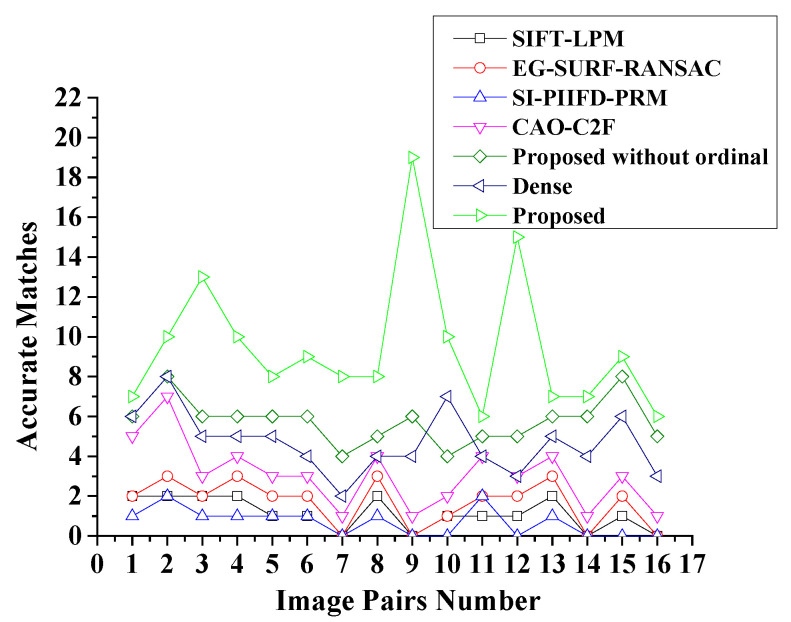
The number of accurate matches based on the seven considered methods.

Moreover, the Precision–Recall curves of the considered algorithms are shown in Figure 13. It can be seen that GOCAO-R2F has the highest average precision among the seven considered algorithms, due to rich contour and texture features, robust main orientation description of GOCAO. Meanwhile, GOCAO-R2F achieves the most matched feature points in each multi-source porcine body image pair, as shown in Figure 14.

To prove the real-time operation of the proposed registration method, the average running times of the considered visible and infrared registration methods are shown in Table 2, and the sizes of multi-source porcine body images are the same in the processing of each run. The results can validate that GOCAO-R2F has great advantages in efficiency among the considered state-of-the-art smart visible and infrared image registration methods, except CAO-C2F and Dense. The reason for this phenomenon may be that the proposed method takes a long time to represent multi-source porcine body image features, which reduces the overall efficiency of the registration algorithm.

### 3.3. The Porcine Body Multi-Feature Representation

To prove the accuracy of the proposed registration algorithm for porcine body multi-feature representation, visible and infrared porcine body image pairs are consistent with Section 3.2.

**Figure 15:**
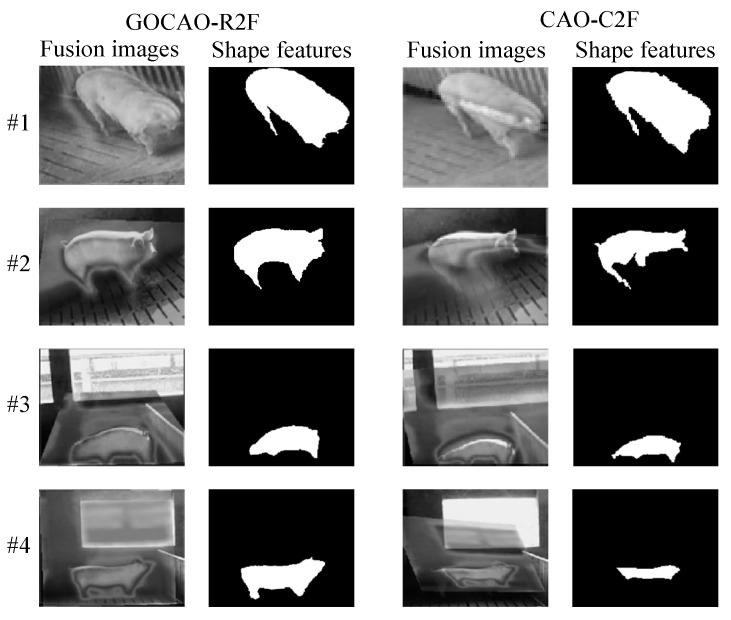
The porcine body shape features under variable illumination conditions.

**Figure 16:**
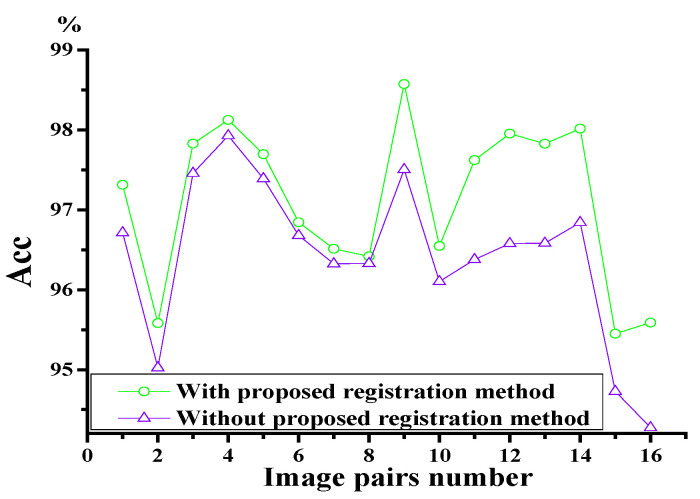
The average accuracy of porcine body shape features with different registration methods.

Because CAO-C2F has the best registration performance among the five considered comparative methods, which is proven in Figure 12, the extracted shape features are only compared with those of CAO-C2F. In this paper, MGANFuse [28] is used to obtain multi-source porcine body fusion images and shape features with variable illumination conditions, which are shown in Figure 15. To validate the detection performance of porcine body shape features with the presented registration algorithm, an average accuracy is adopted to measure porcine body shape features, as shown in Figure 16. It can be seen that the representation method based on the proposed registration model can more effectively represent porcine body shape features, especially in poor illumination.

**Figure 17:**
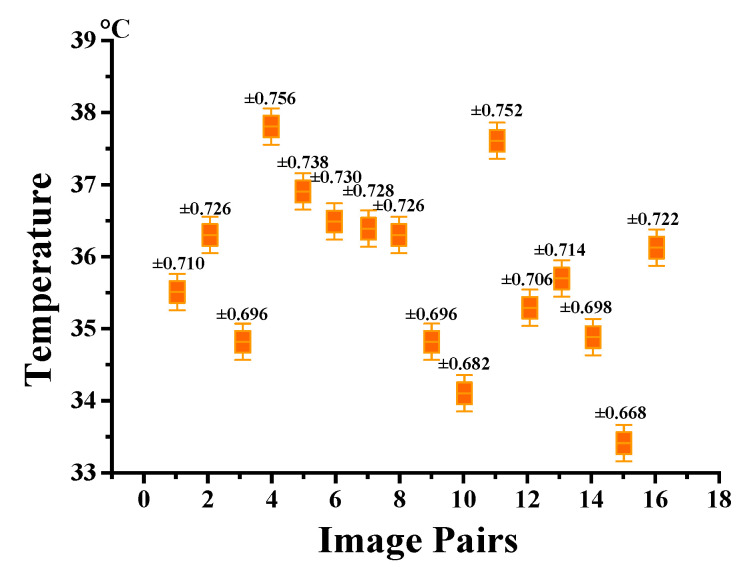
The porcine body temperature features under variable illumination conditions.

Then, the temperature features are extracted in view of shape features [35], and the porcine body temperature features are obtained and shown in Figure 17. It can be seen that due to a 2% error of FLIR C2, the obtained temperature features have a certain range of error, and the highest temperature features with floating error are all in the normal range of animal temperature.

## 4. Discussion

**Figure 18:**
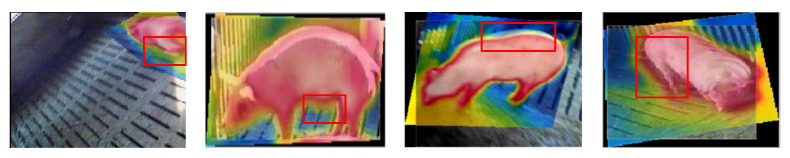
The examples of bad situations.

The limitation of the proposed multi-source porcine body image registration method is greatly affected by bright illuminations, which reduce the accuracy of multi-source porcine body image matching, as shown in Figure 18. From the figure, there are slight deviations in the matching of porcine body shape edge features under brighter illumination, which can affect the accuracy of porcine body shape feature extraction. The possible reason is that there are too many feature points in the visible porcine body image under brighter illumination conditions, which can easily cause mismatching in visible and infrared porcine body image registration. The possible solution is whether to replace the Ordinal operation with the Local Binary operation [36], which is more robust to variable illumination conditions and can reduce the effect of bright illumination.

## 5. Conclusions

A novel visible and infrared porcine body image registration algorithm called GOCAO-R2F is presented for multi-feature detection. Firstly, porcine body contour features are acquired by a Gabor filter with variable scales and an ordinal operation. Secondly, feature points in contour features are obtained via CSS, and the main orientations of each feature point are determined using the GOCAO. Thirdly, modified SIFT features are received on the main orientation and registered via R2F. Finally, the porcine body multi-feature is represented in view of registration results. Experimental results show that the presented registration algorithm accurately matches visible and infrared images for porcine body multi-feature representation and is capable of achieving lower average root-mean-square errors than current registration algorithms.

This work mainly focuses on registering multi-source porcine body images in variable illumination conditions. The limited number of multi-source porcine body image pairs and the limited automation performance of the presented registration method can reduce the adaptability of the algorithm. Future studies should focus on robust feature representation of multi-source porcine body image and an adaptive registration model for visible and infrared porcine body images under occlusion and noisy environments, which can improve the robustness and accuracy of the porcine body multi-feature representation method in view of multi-source image registration. In addition, it is necessary to continuously improve the multi-source porcine body image database.

## Figures and Tables

**Table 1:** Main technical indicators of FLIR C2.

Technical Indicators	Descriptions	Technical Indicators	Descriptions
Resolution of infrared images	80 × 60 ppi	Resolution of visible images	640 × 480 ppi
Measuring temperature range	−10–+150 °C	Focus form	Fixed focus
Precision	Error is 2%		

**Table 2:** The average running times of multi-source image registration methods.

	Registration Methods					
	SIFT-LPM	SI-PIIFD-LPM	EG-SURF-RANSAC	CAO-C2F	Dense	Proposed
Time(s)	3.752	2.531	2.354	1.347	1.263	2.085

## Data Availability

Data will be made available on request.
